# A novel video-tracking system to quantify the behaviour of nocturnal mosquitoes attacking human hosts in the field

**DOI:** 10.1098/rsif.2015.0974

**Published:** 2016-04

**Authors:** N. C. Angarita-Jaimes, J. E. A. Parker, M. Abe, F. Mashauri, J. Martine, C. E. Towers, P. J. McCall, D. P. Towers

**Affiliations:** 1 Optical Engineering Group, School of Engineering, University of Warwick, Coventry CV4 7AL, UK; 2 Vector Biology Department, Liverpool School of Tropical Medicine, Pembroke Place, Liverpool L3 5QA, UK; 3 National Institute for Medical Research, Mwanza Medical Research Centre, PO Box 1462, Mwanza, Tanzania

**Keywords:** mosquito, tracking, imaging, behaviour, algorithm, malaria

## Abstract

Many vectors of malaria and other infections spend most of their adult life within human homes, the environment where they bloodfeed and rest, and where control has been most successful. Yet, knowledge of peri-domestic mosquito behaviour is limited, particularly how mosquitoes find and attack human hosts or how insecticides impact on behaviour. This is partly because technology for tracking mosquitoes in their natural habitats, traditional dwellings in disease-endemic countries, has never been available. We describe a sensing device that enables observation and recording of nocturnal mosquitoes attacking humans with or without a bed net, in the laboratory and in rural Africa. The device addresses requirements for sub-millimetre resolution over a 2.0 × 1.2 × 2.0 m volume while using minimum irradiance. Data processing strategies to extract individual mosquito trajectories and algorithms to describe behaviour during host/net interactions are introduced. Results from UK laboratory and Tanzanian field tests showed that *Culex quinquefasciatus* activity was higher and focused on the bed net roof when a human host was present, in colonized and wild populations. Both *C. quinquefasciatus* and *Anopheles gambiae* exhibited similar behavioural modes, with average flight velocities varying by less than 10%. The system offers considerable potential for investigations in vector biology and many other fields.

## 1. Introduction

Many of the major arthropod vectors of disease live in close association with their human hosts. Worldwide, numerous vectors of malaria, dengue, yellow fever, filariasis, leishmaniasis and Chagas disease spend much of their adult life resting and bloodfeeding within human habitations. This is a vulnerable point in their life cycle, and targeting adult vectors in the home is an effective method of interrupting disease transmission. Knowledge of vector behaviour in and around human housing is often limited to generalities or simple endpoint events like locations and times of entering, arrival at the host, resting or exiting. A better understanding of vector movement within domestic dwellings and their interaction with control interventions such as bed nets could enable rational design of intervention tools, but technological challenges have hindered progress.

Many existing diagnostics to quantify mosquito behaviour are based on assessing their response to stimuli, e.g. odours or insecticides, giving information on attraction or deterrent effect [1–5]. The test may be set up to collect mosquitoes in traps positioned upwind of the odour [2] or use lethal electrocution nets to capture mosquitoes responding in a particular direction [1]. Semi-field experiments use a netted volume in order to define the mosquito population studied while operating in a natural environment [5]. Mosquitoes may also be labelled with fluorescent dye or powder for manual identification but the labels can affect mosquito survival [6].

Imaging techniques have been used to obtain more detailed information on mosquito behaviour. Wind tunnel studies with transparent-walled test sections enable imaging with single or multiple cameras and hence two- or three-dimensional measurement of mosquito positions resolved in time can be obtained. Investigations of insecticide repellency, sensitivity to carbon dioxide, odour and heat stimuli have been conducted in both two [7,8] and three dimensions [7,9,10]. However, despite their undoubted value, such studies are unable to observe mosquito responses to a complete human host or to capture and analyse approach and attack behaviours at control interventions such as bed nets. Furthermore, the previously cited wind tunnel studies were limited to analysing single mosquito trajectories, from a maximum of four in the field of view, and for up to 15 min. Recently, sophisticated laboratory systems have tracked 20 diurnally active *Aedes aegypti* mosquitoes for 3 h [11,12], and three fruit flies for 1 h [13] but their complexity (four to six cameras and one to six computers) limit the system's potential for field studies while the illumination method in [11,12] constrained the field of view to wind tunnel scales of 1.2 × 0.3 × 0.3 m and to a 10 cm dome in [13]. The flight chambers in laboratory experiments typically provide multiple view optical access of the relevant volume, often the entire flight domain. In such geometries, temporary occlusion of insect positions occurs mainly due to overlapping images from multiple insects and suitable tracking algorithms, for example, based on Kalman filters [13,14], are applied to obtain smooth flight tracks. The biological events to be studied here present different challenges with higher probabilities of occlusion from semi-fixed objects such as the bednet and its supporting strings, folds in the net causing regions of varying intensity and multiple flying mosquitoes, all of which contribute to loss of mosquito position data in single or multiple frames.

A significant advance towards the observation of natural behaviours in the field were studies of mosquito mating swarms using the setting sun as a back-light for a stereo camera pair [15,16]. Individual mating events could be identified from the three-dimensional reconstructed tracks. Segmentation of mosquito images in individual image frames and the tracking of these positions could be done automatically using bespoke algorithms but required validation and approximately 30 min manual assessment for a 60 s track (250 frames). Individual tracks of up to 60 s duration were recorded and approximately 62 000 mosquito positions obtained in total from male mosquitoes in 10 swarms—the state of the art in 2014 [16]. However, the imaging approach cannot be translated to study nocturnal behaviours (where no ambient lighting may exist) and within domestic dwellings—key targets that must be addressed to study behaviour at the host interface. Other field studies have examined tracks of bats, making use of three infrared cameras to track clouds of bats by thermal sensing [14]. The placement of the third camera allowed better three-dimensional coordinate reconstruction when occlusion occurred in one camera view and coupled with the data processing strategies developed to merge the data from the other views. Other fields of study have also targeted imaging within domestic dwellings, e.g. to quantify airflow distributions in order to minimize energy consumption in buildings by recording the movement of flow tracers [17]. Typically, front lighting is used with multiple bulbs of more than 100 W each to illuminate helium-filled soap bubble tracers of 1–4 mm diameter. Such illumination would affect nocturnal mosquito behaviour [18]. The image processing of airflow tracer data is also more straightforward as domestic household flows exhibit limited spatial gradients and hence processing algorithms can use both spatial and temporal consistency to quantify the flow field [19], whereas multiple mosquitoes behave independently of each other in most activities.

The primary goal in developing the tracking system reported here was to investigate detailed aspects of host-seeking behaviour of mosquitoes at a human-baited bed net. The first biological findings from laboratory-based experiments have been reported [20], and here, we describe the design of the imaging system and quantify its performance. We also address the analysis of long time series, up to 1 h, 50 frames per second (fps) image sequences of natural mosquito populations with tens of mosquitoes present per frame necessitating increased automation and robustness of the data processing algorithms. To demonstrate the versatility of the approach, experimental data are given from both the laboratory in UK and the field in Tanzania, with *Culex quinquefasciatus*, a vector and cosmotropical nuisance mosquito and *Anopheles gambiae*, a primary malaria vector in sub-Saharan Africa.

## 2. Material and methods

### 2.1. Imaging system

**Figure 1.**
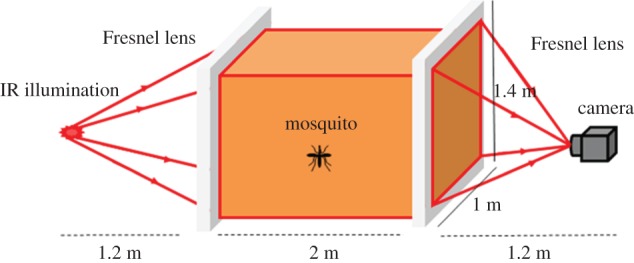
Schematic of the back-lit imaging set-up. The system consists of two Fresnel lenses with focal length 1.2 m (lens aperture = 1.0 × 1.4 m), an imaging lens (focal length 12.5 mm) and a high-power LED at 850 nm. The field of view of the imaging lens at the Fresnel focal length distance is 1.2 m × 1.2 m, and the actual volume imaged is 1 × 1.2 × 2 m (width by height by depth).

Tracking mosquitoes as they fly within the space surrounding a bed net requires the imaging of an extended volume. Conventional diffuse imaging spreads the illumination over the target and captures the light scattered with a lens and camera. For metre-scale fields of view, this approach is optically inefficient as the scattering process intrinsically gives a loss by a factor of more than 10 000 : 1 (1 m^2^ compared to a lens aperture of less than 100 mm^2^). A further drawback of conventional imaging is that increasing the depth of field by decreasing the aperture leads to further reductions in optical efficiency and concomitant worsening of the lateral resolution. Back-lit imaging offers the opportunity to resolve both optical efficiency and depth of field issues [21]. Figure 1 illustrates the approach used in this study. An infrared (IR) LED (OSRAM SFH 4230) at 850 nm provides up to 440 mW of illumination in which the FWHM angle is 120°. This wavelength falls within the ‘mosquito-blind’ near-IR region [18]. Addition of a diffuser helps to homogenize the illumination. A large aperture Fresnel lens enables the illumination to be formed into an approximately collimated beam. At the other end of the measurement volume, a matched Fresnel lens combines with the camera's imaging lens in a telecentric arrangement, and mosquitoes are visualized as shadows along light rays parallel to the optical axis. The effective field of view (EFOV) of the system is 1.0 × 1.2 m, determined by the focal length of the Fresnel lenses and the imaging lens and the Fresnel lens aperture. Optical system efficiency allows reduction of the camera's exposure time to typically 3000 µs, delivering sharp images of mosquitoes at maximum flight velocities.

#### 2.1.1. Resolution achieved in a large field of view system: two dimensions

The back-lit approach reduces the three-dimensional measurement volume to a two-dimensional image. The depth of the imaging volume was limited in order to maintain sufficient contrast in the mosquito images for robust segmentation. Mosquitoes are large compared with the wavelength of light and therefore the diffraction pattern in the far field consists of a shadow of slowly increasing diameter with distance from the object [22]. The ability to resolve such shadow images was evaluated experimentally by placing nine different objects (widths from 280 to 2500 µm) at different distances along the optical axis. The image width obtained was quantified by fitting a Gaussian function to the intensity profile, 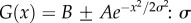 of the fitted function is the standard deviation and relates to the width of the object, *B* represents the image background and *A* the maximum intensity of the profile. The variation of the width measurements, characterized by the standard deviation at the different distances, was estimated.

The system magnification was determined by means of a calibration plate and an average taken from different positions within the imaging volume.

### 2.2. Video-tracking system

#### 2.2.1. Recording system

To characterize mosquito activity at a bednet, two of the back-lit imaging set-ups described above were operated in parallel giving a total field of view of 2 × 1.2 m with 2.0 m in depth, i.e. sufficient to contain a prone human host within a bednet as well as the space immediately surrounding them. Enhanced near IR (NIR) sensitivity cameras were used to suit the illumination at 850 nm from the IR LEDs.

#### 2.2.2. Active capture functionality

**Figure 2.**
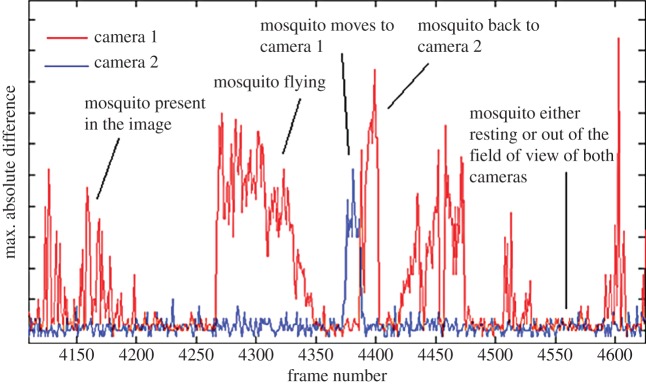
Changes in the maximum absolute difference (in grey levels) between consecutive frames as a function of frame number (time). The high values correspond to mosquito movement. Two cameras were set up as shown in figure 1, with views at the upper/head and lower/foot regions of the human ‘bait’ (e.g. figure 8). A threshold can be defined to record only those images with mosquito, or other movement.

Initial observations revealed that periods of mosquito activity were interspersed with lengthy periods of inactivity when mosquitoes rested or were not present within the observable view. To mitigate the amount of data requiring processing, an algorithm for intelligent data capture was developed such that only images with moving mosquitoes present were recorded. The algorithm operates in real time by finding the absolute maximum per pixel greyscale difference between consecutive image frames, termed the *maximum absolute difference*. There is sufficient sensitivity and low image noise to detect the small intensity changes produced by a flying mosquito and the calculation is simple enough to run in real time and at high frame rates, although image capture is also triggered by other insects and by movements of the volunteer (human bait) or the bednet. The algorithm is implemented as a module within StreamPix 5 and user control of the threshold for image capture, is typically set between 4 and 10 grey levels. Figure 2 illustrates typical data for the maximum absolute difference recorded simultaneously from two cameras with mosquitoes entering and leaving the fields of view.

### 2.3. Automatic detection of mosquito position

**Figure 3.**
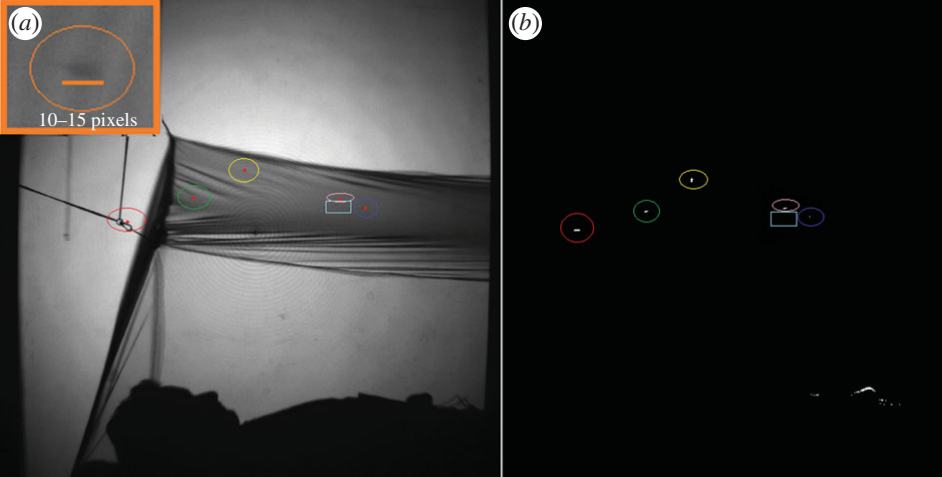
(*a*) Recorded frame with six mosquitoes present. The mosquito images are small compared with the whole image size, as illustrated by the close up of a mosquito image shown in the insert (top left). (*b*) The binarized image of (*a*) after sequential frame subtraction and initial filtering. Human movement around the lower abdomen, where the hands were resting, was picked up by the algorithm and subsequently removed. The centre positions of five out of the six mosquitoes are denoted by the dots in (*b*), the 6th mosquito, denoted by the rectangular box, is immobile on the bednet.

By definition, the imaging of mosquitoes over a large field of view results in images whereby the object of primary interest, the mosquito, is relatively small and with limited contrast given the imaging conditions (figure 3, inset). Hence, a strategy based on motion was used to identify and segment individual mosquitoes within the image.

#### 2.3.1. Subtraction of consecutive images

The segmentation algorithm starts by finding the per pixel difference between successive frames (figure 3*a*,*b*), retaining the positive values only.

#### 2.3.2. Binarization of difference image

‘Changed’ regions in the image are associated with flying mosquitoes (or another moving object): thus the difference image is binarized by assigning ‘1’ to those pixels above a user-defined threshold and ‘0’ to those below. This threshold is estimated for each pair of images based on the signal-to-noise ratio (SNR) and a global minimum value is set to account for images where there are no moving objects while eliminating areas of noise due to uneven illumination. A first filtering process is performed at this stage, where any objects outside a predefined area range, 6–400 pixels, are classified as ‘noise’ and removed.

#### 2.3.3. Removal of human/bednet movement

Movement by the human volunteers or bednet varies in magnitude and may not be completely removed by the initial area-based filter. At the frame rates used, up to 50 fps, the ‘changed’ regions of the volunteer correspond to its contour (figure 3*b*). Hence, by identifying the human silhouette and net structure, any positions within a predefined distance, up to 15 pixels, can be removed. This process is applied only when necessary, by identifying the frame numbers where such movement is observed.

#### 2.3.4. Identification of mosquito centres

An intensity-weighted centre of mass calculation is used to calculate the coordinates of the segmented objects corresponding to individual mosquitoes.

#### 2.3.5. Performance assessment of segmentation algorithm

To assess the performance of the algorithm, a set of artificial mosquito images were generated from fitted two-dimensional Gaussian intensity models. In simulations, pairs of frames were generated with increasing mosquito translation in the second image under the assumption that mosquito orientation does not change between a pair of images (20 ms). To assess algorithm performance and accumulate statistical data, mosquitoes of two sizes were generated (within the range observed in the recordings) and 100 Monte Carlo simulations randomly selecting the location of the mosquito in the first frame were performed. The root mean square (RMS) error in the mosquito centre positions were compared to the known mosquito centre from the simulation settings for each separation, and averaged across the different locations.

### 2.4. Linking of mosquito positions into individual trajectories

The spatially and temporally resolved set of mosquito centre coordinates was formed into trajectories from which individual mosquito flying paths were determined. The tracking algorithm was written to handle both fast flights above the net, and trajectories where mosquitoes moved slowly across or rested on the bednet with the expectation that only a few mosquitoes are present in any (*x, y*) image region and point in time. Since the segmentation algorithm is based on motion, the positions of stationary mosquitoes would not be detected. This was managed at the tracking stage and is similar to the issue of missing mosquito positions in individual or sequences of frames that can occur due to occlusions from the net or other objects. With reference to previous work on multi-view tracking it was identified by Wu *et al*. [14] that single-view two-dimensional tracking is particularly challenging with large numbers of objects or with a high probability of occlusion.

The assignment of mosquito links between images is based on finding the most likely position in the next frame within a predefined search distance determined by the maximum mosquito velocity. In the approach adopted, multiple potential matches are sorted by maximizing the temporal coherence between the track belonging to the mosquito up to the current frame and the possible assignments found in the next one. In this way, merges and splits are also assigned, i.e.: when the paths of two closely spaced mosquitoes merge into a single trajectory because they temporarily occluded each other, one mosquito rests or when the mosquito could not be detected around dark shaded areas of the field of view (net boundaries, flight directly behind or in front of the human). The assumption is that individual mosquito tracks show a greater level of continuity than any other mosquito's path. To avoid creating broken tracks as a result of such occlusions, during tracking, the search radius was set to be large enough to link tracks moving across narrow occluded areas (boundaries of the LLIN and the string supporting it). Furthermore, tracking parameters permitted gap closing over brief periods during which mosquitoes were not visible [5]. In post-processing steps, trajectories broken by occlusion can be linked across longer distances and longer wait times. Finally, where appropriate, broken tracks may be linked manually by the user.

**Figure 4.**
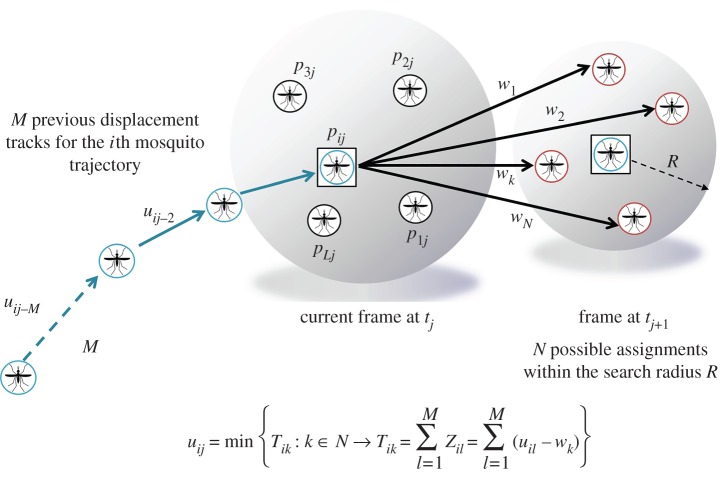
For each mosquito in the current frame, the new assignment *u_ij_* is estimated by finding the link that maximizes the track temporal smoothness. Note that *T_ik_* takes into account both the magnitude and the direction of the movement; hence by minimizing this metric, the most likely assignment can be found. A trajectory is defined as ‘terminated’ when its last ending point is far from the edge of the field of view. Similarly, a trajectory is defined as ‘initiated’ if it starts far from any of the entry points.

A detailed description of the greedy algorithm developed to solve the tracking problem is given below, the notation is defined in table 1 and the problem is illustrated in figure 4.

(1) In the current frame *j* at each mosquito position *p_ij_*, delimit a search radius *R* within which the mosquito is likely to be found in the next frame *j* + 1. *N* is defined as the number of possible assignments for the *i*th mosquito in the *j* + 1 frame.
(2) Determine the most likely displacement vector *u_ij_* by looking at the temporal consistency of mosquito displacements. Calculate the cost of each possible assignment by estimating their temporal coherence: take the *M* previous frames of the *i*th mosquito trajectory up to frame *j* and estimate *T_ik_* (table 1, no. 10) for all *N* potential matches (***w****_k_*). Select the link with the best temporal smoothness, i.e. the one with the minimum *T_ik_*:
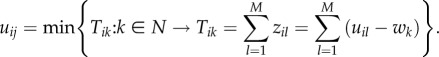
Confirm the assignment by checking if *T_ik_* is below a predefined threshold.
(3) Keep the last known position of any unpaired mosquitoes for a predefined number of frames. This is to deal with resting mosquitoes that will eventually start moving or mosquito images that had become occluded by the bednet so that the trajectory can be resumed. If however, the mosquito cannot be re-assigned soon enough, the current trajectory is terminated and when movement resumes then a new trajectory is initiated.
(4) After finding the assignments for all mosquitoes in the current frame *t_j_*, update the trajectories of the current frame, so that these can be used for the estimation of the temporal coherence functions at the next frame, and so on.
(5) After completing initial pairing for all frames, check for terminated trajectories, i.e. trajectories with ending points far from the edge of the field of view. Evaluate possible connections to ‘initiated tracks’, i.e.: trajectories with starting points far from the edge of the field of view, using the consistency check (2 above) and if *T_ik_* is below the predefined threshold then ‘join’ the trajectories.
(6) Validate trajectories by setting a minimum number of frames where an individual mosquito is required to be present and check for temporal consistency (smoothness) of the complete track. This avoids linking non-mosquito features that can sporadically appear due to noise.
(7) Check for trajectory connections between the two parallel camera views: identify end and start points of trajectories within a specified distance of the common border between the two camera images. Use the consistency test from 2 above to identify trajectories that should be connected.

**Table 1.** Parameters, notation and calculations used for estimation of mosquito flight tracks and behaviour analyses.

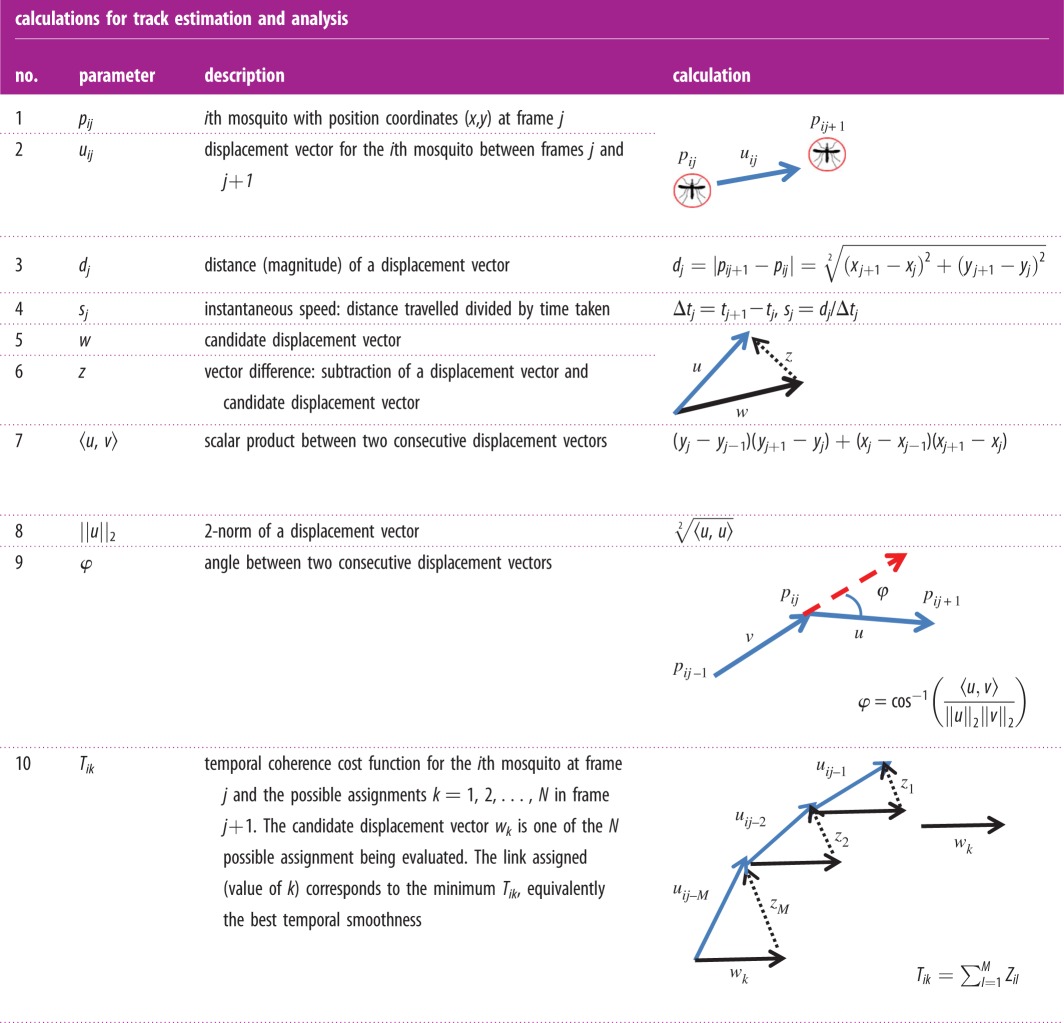

#### 2.4.1. Tracking algorithm and performance assessment

To assess the results of the tracking algorithm a number of performance metrics are defined below. The parameters estimated from the initial tracking (stages 1–4) are:

— number of initial tracks (without reconnections);
— number of mosquito positions along a track; and
— number of tracks with long gaps where the track identity was retained (expressed as a percentage) and the average number of frames per gap.

After checking for unconnected tracks (trajectories that terminated/started far from the edge of the field of view) and making connections where possible (stages 5–7), the final set of tracks is obtained. The Track Fragmentation (TF) metric quantifies how well the mosquito identity is maintained (adapted from [13]). If *T_ij_* is the total number of tracks and *U_ij_* is the number of tracks that could not be connected, i.e. mainly those that terminated or were initiated at the bednet, then TF can be defined as:
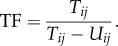
The closer TF is to unity, that is the smaller *U_ij_*, the more likely that a single trajectory was used to describe a mosquito path, and therefore preserving its identity in time.

### 2.5. Classification of mosquito behavioural modes

Mosquito tracks were classified into four flight types, termed behavioural modes, quantified and distinguished by extent of net contact and track velocity, as defined by [20].

(1) Swooping: flights where mosquitoes made no contact with the bed net or the host during the entire trajectory.
(2) Visiting: tracks consisting of long periods of flight punctuated by infrequent contact (visits) with the net or host. Visits were characterized by at least one contact point, or sharp turn of 80° or more, on the net or host surface. Where multiple contacts occurred, the minimum interval between them was 0.4 s and we assumed that the mosquito continued to fly before and after each contact.
(3) Bouncing: tracks where the mosquito made repetitive contacts with the net or host, at intervals of less than 0.4 s. This category included periods of ‘walking’, where mosquitoes appeared to stop flying and stayed in contact with the substrate but without being static.
(4) Resting: tracks where mosquitoes were completely static, or the instantaneous speed of the mosquito was less than 1.33 mm s^−1^. The latter condition was included to allow for missing positions within tracks. Mosquitoes were assumed to be in constant contact with the bed net or host.

Modes 2–4 were not exclusive and a single trajectory potentially could display different modes at different times, e.g. visiting followed by bouncing and finally, resting.

#### 2.5.1. Defining and quantifying contact with a bed net

Bed net surfaces were identified by manually selecting the net corner points in an image and classifying each region as outside or inside the net. Activity was allocated to a region based on the coordinates of the mosquito, and whether the mosquito contacted (behaviour modes 2–4) or was swooping in front or behind of the net (behaviour mode 1).

Contact points during flight were defined as sharp turning points that occurred at any net surface. Position vectors were estimated from consecutive points in a trajectory and a sharp turn was defined by the angle formed by adjacent vectors (table 1, rows 2 and 9). This angular value ranged from 0 (flying straight on) to 180° (turning back to the previous position). After analysis of the range of angles present, 80° was chosen as the minimum angle (threshold) for classification as net contact.

**Figure 5.**
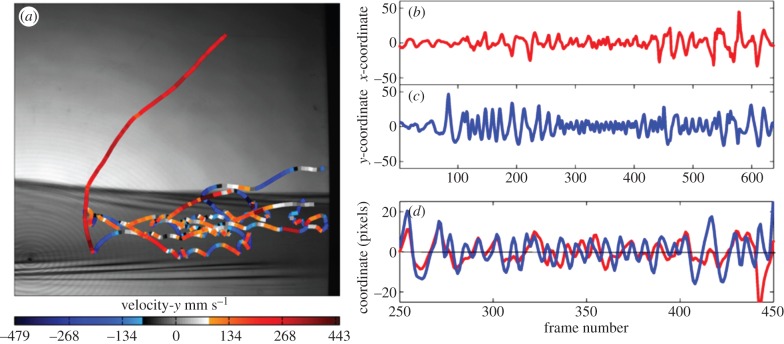
Example of a bouncing trajectory. (*a*) The track has been colour-coded according to the *y*-axis velocity component; (*b*) the *x*-coordinates and (*c*) *y*-coordinates, showing both after bandpass filtering; (*d*) *x-* and *y*-coordinates of the bouncing segment of the trajectory, with the zero crossings between positive and negative peaks indicating contacts with the net.

However, during repetitive attacks, e.g. bouncing mode, not all net contacts are characterized by such sharp angles, and a second set of contacts was identified using variations in the horizontal (*x*) and vertical (*y*) trajectory coordinates. In general, bounces can be defined by semi-periodic changes in the *x-* and *y*-positions; thus after removing the high-frequency and the low-frequency underlying components such that *x-* and *y*-coordinates of each track were centred around a zero mean (bandpass Butterworth filter with cut-off frequency of 1.5 and 15 Hz), bounces and therefore contacts, were characterized by finding the zero crossings between positive and negative peaks (figure 5). The bouncing trajectory in figure 5*a* is colour coded by the vertical velocity component; the colour mapping is designed to highlight the difference between upwards (shades of blue) and downwards (shades of red) movement [23]. The bandpass filtered *x-* and *y*-coordinates are given in figure 5*b*–*d*, with the final number of net contacts derived from the combined set of zero crossings after validation by minimum and maximum temporal limits between contacts.

Other contacts with the net, walking, could only be detected by the sharp angles that adjacent vectors formed, given the unpredictable nature of these movements. Hence, segments of trajectories in walking mode were defined by setting a lower angle threshold at 70° between consecutive displacement vectors, with the additional constraints that walking could only occur on the net and the spacing of the contacts was less than that defined for bouncing.

### 2.6. Experimental procedures

#### 2.6.1. Laboratory tests

*Culex quinquefasciatus* Recife strain and *A. gambiae sensu stricto* Kisumu strain were obtained from colonies at the Liverpool School of Tropical Medicine (LSTM). Tests were carried out on unfed adult females that had the opportunity to mate, 3–5 days post-eclosion, in a large (5.6 × 3.6 m in area 2.3 m high) dedicated insectary at LSTM [20].

#### 2.6.2. Field tests

Field tests were carried out in an experimental hut at a rural location in Kayenze, near Mwanza, northern Tanzania. Adult *C. quinquefasciatus* were reared in insectaries at the National Institute for Medical Research (NIMR) from immature stages collected in local breeding sites, identified morphologically (R. B. Highton 1983, unpublished data) and tested between 21.00 and 22.30 h.

#### 2.6.3. Bed nets

**Figure 6.**
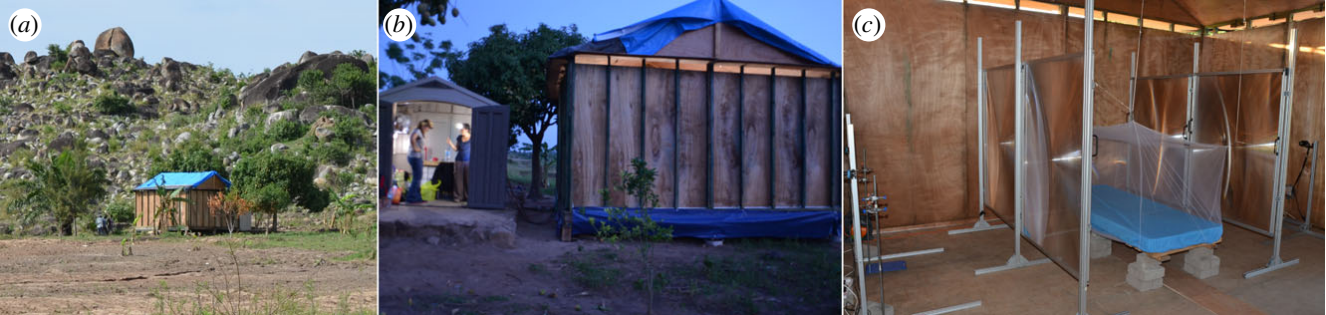
Field studies were undertaken at a rural location near Mwanza, Tanzania. (*a*) The experimental huts were erected approximately 500 m from known natural mosquito breeding sites, and from the nearest human and animal housing. (*b*) The small hut housed the recording unit from where the observer operated the cameras of the tracking system as they recorded mosquito activity in the large hut. (*c*) The complete tracking system showing the LEDs on the left, the paired Fresnel lenses on either side of the bed and bed net and cameras on the right.

Bed nets were assembled from untreated polyester and hung with the top roof tilted on its long axis (figure 6) and tailored to fit the mattress tautly, eliminating wrinkles or folds that could have obscured mosquito detection [20]. Human volunteers, recruited from the research team and local community, lay on their backs on a mattress and were clothed but barefoot and asked to remain still to avoid generating spurious tracks.

#### 2.6.4. Experimental protocols

Laboratory studies used 25 mosquitoes per test, in six and 10 tests for *C. quinquefasciatus* and *A. gambiae* respectively. In addition, three control tests (i.e. an untreated bednet with no human bait present) were also carried out with *C. quinquefasciatus*.

Mosquitoes were starved of sugar and water for 4–6 h before tests and introduced into the experimental room in a 220 ml paper cup suspended 2 m high on the wall, 1 h prior to testing, when the volunteer also entered the net and the room was shut to allow for acclimatization. A cord enabled the mosquitoes to be released from outside the test facility. At the end of 1 h of recording, mosquitoes in the room were collected with aspirators.

#### 2.6.5. Recording system

Tests were carried out in the dark (insectary) and at night (field) with artificial lighting provided by the imaging system's IR LEDs alone. Two Norpix recording systems (hardware and software) were used, each with StreamPix software to provide live previews and control recording of up to two cameras. Enhanced NIR sensitivity Baumer HXC40NIR cameras offering 2048 × 2048 pixels and up to 180 fps were selected with full Camera Link interfaces. Each image capture PC, i7 3.4 GHz, 8 GB RAM, was equipped with 20 terabytes (TB) of storage, two Silicon Software interface cards to support the two Baumer cameras and running StreamPix 5 software for data capture and storage. The system was capable of recording 5.5 h of continuous data from the two cameras at 100 fps. At a capture rate of 50 fps, the system typically generated 1.4 TB data per hour.

#### 2.6.6. Data analyses

The tracking algorithms were implemented in MATLAB and consisted of three modules: an automated component that extracted mosquito positions from each frame; a partly-human-supervised component to verify the extracted positions and apply algorithms to remove noisy data (e.g. human/net movement) before automatically linking mosquito trajectories; a third module enabled track editing and automated procedures to reconnect broken trajectories. These trajectories were then further analysed for assignment to behavioural modes and to extract parameters (sections 2.5 and 2.5.1 and [20]).

#### 2.6.7. Statistical analyses

Using SPSS Statistics 21 (IBM), independent sample *t*-tests and generalized linear models with normal distribution were used to compare the velocity distributions between species, activity times, behavioural modes and net region preferences. When values were not normally distributed according to calculations of skewness and kurtosis, data were log transformed. For the velocity vector analysis, equal sample sizes were obtained by randomizing the selection for inclusion in calculations. The size of the smallest group was taken as the limit and the mean and standard deviation of the original and subsampled population compared to verify that the latter was representative of the original set. Given the positive skew of the datasets (figure 10), data were log transformed for further statistical analyses. Results with both transformed and untransformed datasets were similar; thus results from the non-transformed dataset are presented to aid interpretation.

## 3. Results

### 3.1. Validation and performance of the video-tracking system

#### 3.1.1. Resolution achieved in large field of view system: two dimensions

**Figure 7.**
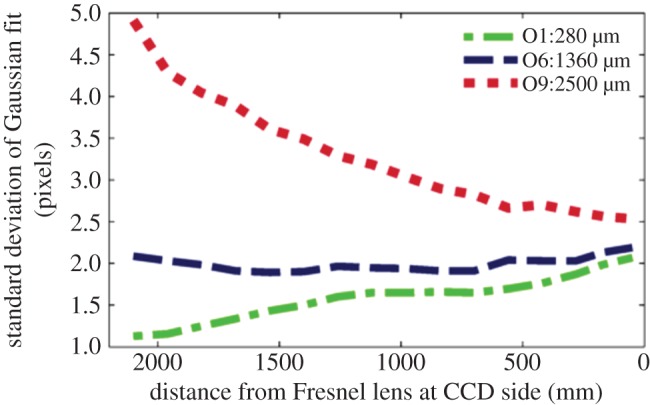
Variation in observed test object size determined from the back-lit imaging arrangement as a function of position along the optical axis, using three test objects of different size (280, 1360 and 2500 µm wide).

Figure 7 summarizes the performance of the system with three test objects of widths 280, 1360 and 2500 µm. The larger width objects produced high-contrast images in which the standard deviation of the Gaussian intensity profile increased with defocus distance (i.e. movement towards the Fresnel lens at the LED). For smaller artefacts, the image contrast was lower and the standard deviation decreased slightly with defocus distance. Similar results were obtained towards the corners of the field of view, where the images exhibited slightly lower contrast.

These data demonstrate the ability of the set-up to resolve mosquito scale objects throughout a measurement volume of 1.0 × 1.2 × 2.0 m per camera. Medium-sized objects, representative of a typical mosquito abdomen 0.4–0.5 mm wide and 1.2–4 mm long, had the most consistent measurements (as indicated by the standard deviation). With an estimated pixel resolution of 0.5 mm in the measurement volume (obtained by averaging the magnification across all points of a calibration grid), the resulting geometric mosquito image was 4–8 pixels in size, but with diffraction and the limitations of the imaging system, became 10–15 pixels (figure 3*a*). Evaluation of and comparison with other camera lens apertures and Fresnel lens separations showed that with the current system, a 2 m separation between the lenses provided an optimal compromise between resolution, available camera lenses and the required measurement volume.

#### 3.1.2. Segmentation and displacement performance

The simulations to quantify the performance of the mosquito segmentation and position measurement algorithms (§2.3) showed two clear trends: the mosquito position error was larger for smaller inter-frame displacements, and there was a dependence on the size of the mosquito. The RMS mosquito position error reduced to a constant level at displacements of approximately 1.3 times the mosquito length, corresponding approximately to Rayleigh's resolution limit. The average position error for inter-frame displacements up to Rayleigh's limit was 0.5 mm (corresponding to 1 pixel), whereas the average overall displacement was 0.12 mm, approximately one-fourth of a pixel. An alternative algorithm to define the mosquito position was evaluated based on locating the peak intensity in the original image according to the location in the difference image (see §2.3). While this approach reduced the displacement at which the position error approached a constant value by 75% and the positional uncertainty at Rayleigh's limit was halved, the algorithm was more susceptible to noise (requiring an additional filtering process) and processing time was doubled. As the position error of the original algorithm applied to the difference image was predominantly in the direction of movement, the overall effect on the trajectory was low, and the original weighted centre of mass approach was preferred to reduce processing time. The automated segmentation process took approximately 13 s per second of recorded footage (from a single camera, code implemented in Matlab).

#### 3.1.3. Validation of the tracking algorithm

Initial analysis of the image sequence obtained showed that 50% of mosquito inter-frame displacements were greater than 10 pixels (5 mm) and 25% were above 22 pixels (11 mm). Thus, the search radius for the initial pairing process was set to 80 pixels (*R* in §2.4). The algorithm was set to wait up to 500 frames (10 s) for a mosquito to re-start moving and maintain its identity and the number of frames for the coherence calculation was set to 10 (*M* in §2.4). Under these conditions, mosquito trajectories frequently had one or two potential assignments at each time step, which could increase to 8 when multiple mosquitoes were present. Merges and splits rarely occurred and were mainly observed on the bednet where either a mosquito was occluded by the bednet or resting. Table 2 shows the performance metrics for the tracking algorithm based on eight experiments in each of which images were captured for 1 h from the two cameras. Experiments 1–6 were conducted in the laboratory in UK, and experiments 7 and 8 were from the field in Tanzania. On average, 1241 mosquito trajectories were extracted initially for a 1 h experiment; in total there were over 3 million tracked mosquito positions for the data summarized in table 2. After applying the track connection process between adjacent camera views (stages 5–7, §2.4.), the number of tracks reduced by 40%, giving a final average of 747 trajectories per experiment.

On average, 378 870 positions of moving mosquitoes were recorded in each experiment, with an average of approximately 300 active positions per track. Following initial tracking, 64% of tracks were found to involve gaps with at least one stationary or occluded frame where the track identity was retained, with an average of 218 of such positions per track. After the connection process (stage 5–7 of the tracking process), the average TF score was 1.4, that is, for every 10 trajectories, three would either terminate at the bednet or between the two fields of view. This value compares well with that reported for a similar metric in Ardekani *et al*. [13] in which there are no obstacles within the measurement volume. During this connection process, a range limit was applied to both the maximum distance between end and start points and the time frame difference (i.e. mosquito velocity), outside which it was assumed that the track did not belong to the same mosquito with the same confidence, and further connections were not attempted. Factors such as fragmented trajectories being removed by the validation process, mosquito images missed in dark bednet areas, mosquito movement being too low to trigger image capture or activity in the blind zone between cameras, all contributed to the final number of unconnected tracks.

### 3.2. Application of the tracking system to quantify *Culex quinquefasciatus* host-seeking behaviour in laboratory tests

The duration of individual flight events or tracks from all laboratory baited tests ranged from 0.38 to 272.2 s, with a mean track length of 23.5 s (1175 frames, including both active and stationary frames). The mean total activity of all 25 mosquitoes within each hour's test (i.e. maximum value of 25 h) was 354 ± 122 min for an average of 1558 ± 660 trajectories. Significantly more (*p* < 0.001) mosquito flight activity involved net contact, either as bouncing (60.5% of flight activity), visiting (29.7%) or resting (5.3%), compared with swooping (4.5%) where no net contact occurred*.* Tracked mosquito flight events are shown in the electronic supplementary material, video, and include trajectories classifiable in each of the four behavioural modes.

Mosquito behaviour was markedly different in the absence of a human host, with a mean total activity of 9 ± 2.5 min recorded for an average of 92 ± 41 trajectories, significantly lower than when nets were baited (independent samples test, *p* < 0.005 for both total activity and number of trajectories). The proportions of time spent in each behavioural mode also were different, with significantly more net contact occurring with a human bait (34 021 ± 1596 recorded contacts, the equivalent of the 25 mosquitoes collectively spending 80 ± 20 min in physical contact with the net) than without (85.6 ± 25 contacts, for a total of 1.5 ± 1.6 min in contact with the net—generalized linear model, *p* < 0.005).

There was no difference in flight speed at unbaited nets (mean velocity 312 ± 12 mm s^−1^) and baited nets (mean 307 ± 16 mm s^−1^; *p* = 0.762). Comparing the average flight velocities in all behavioural modes in human-baited tests (swooping 370 ± 15, visiting 283 ± 11, bouncing 195 ± 14, resting 0.36 ± 0.03 mm s^−1^), with tests using unbaited nets (swooping 342 ± 11, visiting 284 ± 18 mm, bouncing 243 ± 23, resting 0.35 ± 0.19 mm s^−1^), showed that although velocities were significantly different between each behavioural mode (*p* < 0.005), the presence of a bait had no significant effect on flight speed (*p* = 0.252).

**Figure 8.**
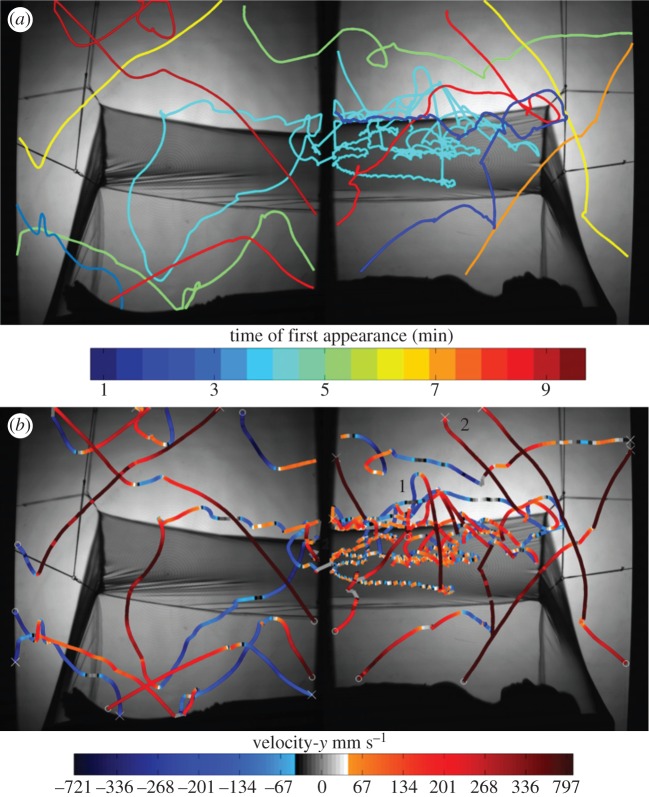
Flight tracks of female *Culex quinquefasciatus* at a human-baited untreated bed net. The sequence includes trajectories classed as swooping, visiting, bouncing or resting behaviour: (*a*) tracks colour-coded according to their first time of appearance (i.e. *N* minutes from the start of the experiment as indicated by the reference bar); (*b*) the same tracks colour-coded by the vertical component of the mosquito velocity; red shows upwards movement, blue downwards and darker colours show higher velocities; the central greyscale represents near zero *y*-axis velocity component; trajectories begin at ‘x’ and end at ‘o’. See also the electronic supplementary material, videos.

The behaviour of individual mosquito trajectories could be classified using the algorithm described in §2.5. Figure 8 illustrates a segment of the mosquito flight events included in the electronic supplementary material, video and shows how flight trajectories may be represented based on a number of different properties. Examining trajectories over time (figure 8*a*) allowed quantification of mosquito persistence levels or decay following intervention and was used by Parker *et al*. [20] to demonstrate how rapidly LLINs exert their effect. Examining tracks based on direction and velocity (figure 8*b*) showed some mosquitoes accelerating rapidly down towards the net before bouncing at lower velocities across the roof (see tracks 1 and 2), as shown by the lighter colour of both red and blue.

**Figure 9.**
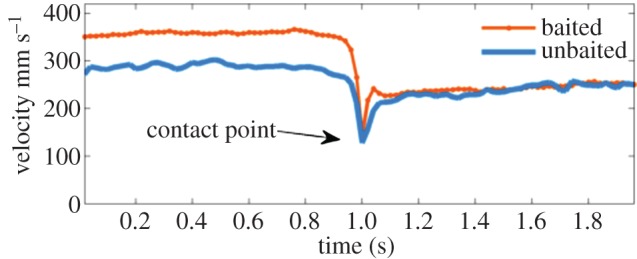
Average velocity of *Culex quinquefasciatus* female mosquitoes, approaching and contacting an untreated bed net in the presence/absence of a host. Figure shows trajectories of mosquitoes contacting the net (*N* = 98 and 1124 for unbaited and baited tests, respectively), where the track positions have been shifted in time such that contact is at 1 s (indicated with arrow).

Quantification of mosquito velocity immediately before and after bednet contact showed that mosquitoes decelerated immediately prior to contact (figure 9), indicating that mosquitoes detected the net and slowed to avoid potentially injurious ‘impact’ at higher speed, as reported previously for *A. gambiae* [20].

### 3.3. Quantifying and comparing behaviour of *Culex quinquefasciatus* and *Anopheles gambiae*

We explored the potential of the tracking system to discriminate between these two mosquito species. Both are nocturnal bloodfeeders, commonly found biting humans simultaneously in Africa where they are disease vectors, and both are important model organisms for many disciplines.

**Figure 10.**
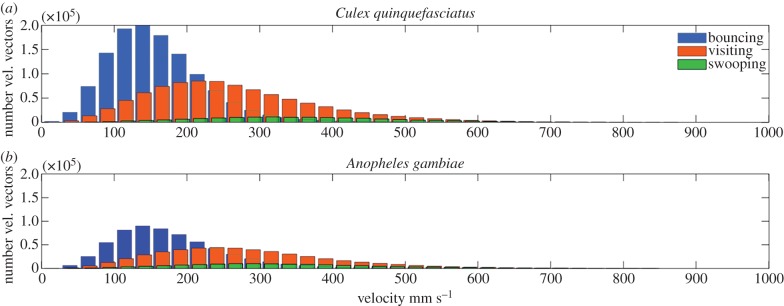
Distribution plot of velocity vectors recorded for different behavioural modes for *Culex quinquefasciatus* (*a*) and *Anopheles gambiae* (*b*) at a human-baited untreated bed net (six and 10 laboratory tests, respectively).

At human-baited bednets, *C. quinquefasciatus* were significantly more active than *A. gambiae* with mean activity totals per test of 354 ± 122 and 129 ± 40 min, and average numbers of trajectories per test of 1558 ± 660 and 544 ± 188, respectively (*p* < 0.001, independent samples test). We also compared the velocities of both species in each behavioural mode as shown in figure 10.

Differences between both species were statistically significant for all behavioural modes (*p* < 0.001, generalized linear model) though not all were in the same direction. Hence, in swooping flight, *C. quinquefasicatus* (mean velocity s.d. 362 ± 148 mm s^−1^) flew faster than *A. gambiae* (342 ± 155) while in visiting and bouncing modes, *A. gambiae* (286 ± 131 and 180 ± 88 mm s^−1^ for visiting and bouncing, respectively) were faster than *C. quinquefasciatus* (268 ± 124 and 162 ± 79 mm s^−1^).

### 3.4. Application of the tracking system in the field

An important requisite of the tracking system was its suitability for deployment in disease-endemic countries to investigate ‘natural’ behaviour of local mosquito populations. When doors and eaves of the experimental hut (figure 6) were left open, a mixed-species population of *Culex*, *Anopheles* and *Mansonia* mosquitoes entered the experimental hut. Hence, to evaluate the system's performance against a single species group, adult *C. quinquefasciatus* reared from larvae harvested from nearby pools were released in to a closed experimental hut, and flights around a human-baited bednet were tracked. Low numbers of mosquitoes at the time of the study restricted tests to two repeats with 20 and 12 mosquitoes.

**Figure 11.**
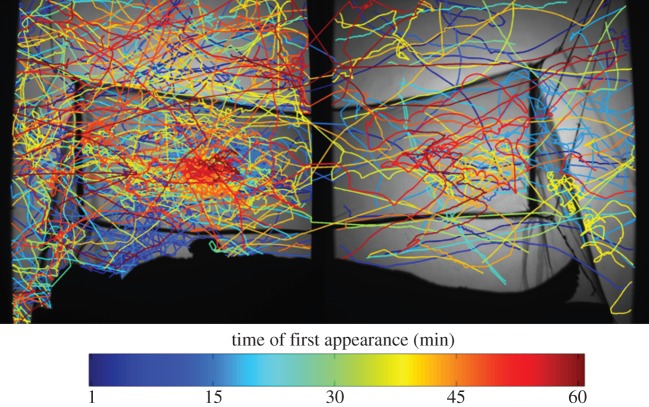
Image of tracks obtained from large field of view back-lit imaging in the experimental hut at a field site in Tanzania, showing flight trajectories from multiple *Culex quinquefasciatus* at a human-occupied untreated bednet. The image contains 414 tracks from 60 min of recording. Each track has a unique numerical identifier, and track start time is colour-coded as indicated by the colour bar (see the electronic supplementary material).

Comparing the images from the field hut in figure 11 with those obtained using the laboratory-based system (figure 8*a*), the field hut image is seen to be slightly inferior, with illumination less uniform across the image. The uneven hut floor of locally sourced timber compromised precise alignment of the Fresnel lenses and camera resulting in darker areas in the images that in turn increased noise levels. The effects of this were manifest in the slightly poorer tracking algorithm performance statistics for the two field experiments (table 2, rows 7 and 8), where the proportion of unconnected tracks averaged 17.4% compared with 10.4% for laboratory experiments. Despite this limitation, the ability of the system to quantify behavioural interactions between mosquitoes at a human-baited bednet was not significantly impaired, and it was possible to extract and analyse mosquito trajectory data at a similar level to that previously reported from the laboratory [20].

**Table 2.** Performance analysis of the tracking algorithm. All percentages were calculated based on the initial number of tracks. The number frames per gap indicates the number of frames where a mosquito was either stationary or occluded and hence not detected by the motion-based segmentation algorithm but has been successfully tracked (retaining mosquito activity). TF indicated if a mosquito path was fragmented into more than one trajectory.

exp. no.	initial number tracks	number mosquito positions	tracks with long gaps (%)	average number frames per gap	number of connections	track fragmentation
1	2495	704 486	72	176	1293	1.45
2	2162	548 188	63	204	1031	1.41
3	862	296 882	62	216	394	1.56
4	1592	705 511	69	220	758	1.44
5	930	263 147	68	187	457	1.44
6	1312	430 305	67	197	650	1.49
7^a^	409	62 911	69	281	210	1.46
8^a^	169	19 538	41	261	39	1.34

^a^Field hut experiments.

### 3.5. Spatial distribution and activity of *Culex quinquefasciatus* at a human-baited bednet

**Figure 12.**
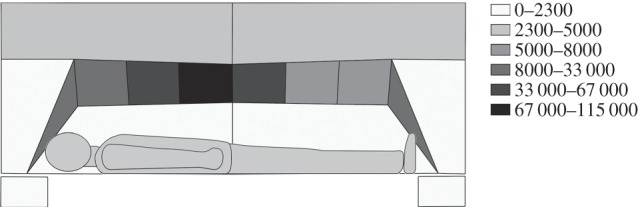
Spatial distribution of mosquito activity for *C. quinquefasciatus* at a human-baited untreated bed net (laboratory tests only). Recorded mosquito tracks were assigned to one of the 16 regions shown: the net surface was divided into 10 regions (six on the roof, two at the sides, two at head/feet); space around the net was divided into six regions: two above the net, two beyond the head and feet and two (shown as boxes beneath the main image) representing the space in front of the net (either upper body/left or lower body/right as viewed in figure) where activity occurred without net contact. Greyscales represent density of activity within a region expressed in seconds per square metre.

The spatial distribution of *C. quinquefasciatus* activity at the surface and surrounding areas of the bed net was mapped by region as shown in figure 12. Analysis of the distribution from the laboratory tests indicated that activity was not randomly distributed across the different regions of the net (random effects generalized linear model *p* < 0.001, *N* = 10), and that highest levels (36.3%) of mosquito activity occurred in the roof area above the lower torso. In fact, 68.3% of all activity occurred within the three net roof regions above the volunteer's head and chest. By contrast, approximately 1% of activity occurred in the two spatial regions at the side of the net (shown as boxes in figure 12).

Analysis of field data also indicated activity to be non-randomly distributed across the net (*p* < 0.009, *N* = 2). Again, mosquitoes showed a preference for the roof of the net above the volunteer's head and chest with 70.4% of activity occurring within these three regions and 47.5% above the lower torso, with 2.2% and 9.8% activity recorded in the two spatial regions at the side of the net.

**Figure 13.**
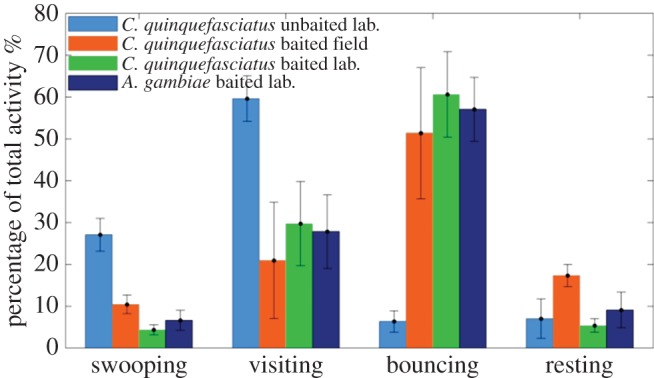
Behaviour of laboratory and wild populations (Mwanza, Tanzania) of different mosquito species at a human-baited untreated bed net, showing the proportion of total activity spent in each behavioural mode. Error bars indicate 1 s.d.

Finally, we compared all the test results from field and laboratory populations of *C. quinquefasciatus* and laboratory population of *A. gambiae,* obtained with the same tracking system by comparing the behavioural mode data from each set of tests (figure 13). Interestingly, results were very consistent: at human-baited nets, there were no differences between the species or populations, with similar proportions of time spent in each behavioural mode when responding to human bait within the bed net (generalized linear model, *p* = 0.936).

## 4. Discussion

The ability of a novel back-lit imaging system to image mosquitoes across an extended volume of 2.0 × 1.2 × 2.0 m is demonstrated. The imaging system is optically efficient, uses low power density illumination in a mosquito-blind region of the spectrum and performed well under very basic field conditions. Additionally, frame difference techniques enabled mosquitoes to be tracked during free flight above, in front of, behind or in contact with a human-baited bednet. The tracking algorithm employed a temporal coherence function in order to cope with background noise levels and movement of other objects, typical of experimental images. Previous tracking techniques based on correlation analysis used the spatial consistency of a group of moving particles; however, given the independent nature of mosquito behaviour and that the images obtained were integrated along the line of sight from a three-dimensional volume, assumptions about spatial coherence were no longer valid, hence the necessity of a new approach. Our complete tracking algorithm uses multiple passes of the data in order to connect tracks where suitable criteria are satisfied, and enables continuity of tracks across multiple camera views. The high frame rates helped minimize the likelihood of incorrect correspondences between mosquito images since the typical mosquito displacement between frames was smaller than the inter-mosquito spacing. The level of automation in the data processing enabled analysis of over 50 times the number of mosquito positions reported previously.

For the first time, populations of important mosquito vectors can be tracked during nocturnal host-seeking for long time periods, under natural conditions in the field. Individual identities are maintained while a mosquito is within the field of view of either camera, enabling simultaneous tracking of multiple mosquitoes. Typically, approximately 1200 tracks were recorded from 25 mosquitoes over a continuous 1-h period with individual tracks up to 300 s in duration.

Examining key behavioural elements in the data presented, *C. quinquefasciatus* activity, presumably in response to the host beneath, was focused at the bed net roof. In this respect, their behavioural responses were similar to those reported for anopheline mosquitoes [24,25] and with the same tracking system [20]. Moreover, responses were remarkably similar in colony and wild mosquito populations, and whether measured under laboratory or field conditions (figures 10 and 12).

As with *A. gambiae* [20], *C. quinquefasciatus* activity was significantly higher in the presence of a human host, particularly in bouncing and resting modes, concurring with hypotheses of mosquito host location behaviour [26,27], with swooping akin to prospection for host cues, while visiting and bouncing indicate direct-oriented responses to host cues, albeit interrupted by the net.

This study did not attempt to use trajectory characteristics to distinguish between *C. quinquefasciatus* and *A. gambiae*, but initial results suggested that potential exists to enable such discrimination in future. Calculations of image size in these tracks found *C. quinquefasciatus* to be approximately 17% larger than *A. gambiae* mosquitoes, and larger insect species generally fly faster [28]. While *C*. *quinquefasciatus* did show higher velocities during swooping mode, *A. gambiae* flew faster in the more tortuous bouncing mode, possibly facilitated by their smaller inertia. Comparison of flight velocities between the two species in each mode, showed up to 10% average overall variation. Efforts to identify reliable methods of discriminating these and other important mosquito genera will continue.

Although at a very early stage, we consider the system's value to vector control to be evident, while additional applications for many lines of basic or applied research are also possible. This tracking system has been used already to describe the detailed mode of action of bed nets [20], and could facilitate studies on behaviour of different mosquito species or forms during responses to insecticides [7,20] or physical barriers [29]. An ultimate goal of such investigations is the development of novel, more effective tools [30] for the prevention of transmission of infections by these important parasites of humans and other animals.

## Supplementary Material

README Supplementary Material

## Supplementary Material

Mosquito flight tracks at a human-baited bed net.

## Supplementary Material

Figure8_velocity_tracks

## Supplementary Material

Figure8_Image

## Supplementary Material

FIgure11_Field_tracks

## Supplementary Material

Figure11_Image

## Supplementary Material

Figure12_Spatial distribution of mosquito activity

## Data accessibility

Data supporting this article have been included in the electronic supplementary material.
