# Supplementary material for: A novel video-tracking system to quantify the behaviour of nocturnal mosquitoes attacking human hosts in the field
Source: J R Soc Interface. 2016 Apr;13(117):20150974. doi: 10.1098/rsif.2015.0974 (PMC4874425; doi:10.1098/rsif.2015.0974)
Supplement: README Supplementary Material [file rsif20150974supp1.pdf]

The following supplementary material has been used to support part of the results presented in the article:

A novel video-tracking system to quantify the behaviour of nocturnal mosquitoes attacking human hosts in the field

N.C. Angarita-Jaimes, J.E.A. Parker, M. Abe , F. Mashauri, J. Martine , C.E. Towers, P.J. McCall2, D.P. Towers

List of files:

Summary_flight_trajectories_Culex.xls:

This file includes a summary of the flight trajectory data recorded for female *Culex quinquefasciatus* at a human-baited untreated bed net. All experiments both in the lab and the field are summarised (one tab per experiment) within the file.
The following information is included per experiment:

Mosquito ID: Number identifying individual mosquito
Trajectory start: time of first appearance (seconds) of each mosquito relative to the start of the recording
Trajectory duration: given in seconds
Distance travelled: total distance travelled by the mosquito in the given trajectory
Speed: Average speed estimated from the average of all the velocity vectors that form the trajectory (given in mm/sec)

Figure8_velocity_tracks.txt

This file provides the complete flight trajectory information presented in Figure 8. The file contains 7 columns defined as follows:

X (pix): X coordinate of the flight trajectory in pixels
Y (pix): Y coordinate of the flight trajectory in pixels
Frame number: frame within the video sequence
Mosquito ID: Number identifying individual mosquitos
Time Stamp: displays the absolute time stamp when the current frame was captured. It is divided in three columns
Time Stamp (1): Time (HH:MM:SS) (for conversion in Matlab use :
Time Stamp (1)/86400 + datenum(1970,1,1)
Time Stamp (2): FFF(msecs) (No conversion required)
Time Stamp (3): UUU(usecs) : No conversion required

Figure8_Image.jpeg

Background image for tracks in Figure 8.

Figure11_field_tracks.txt and Figure11_Image.jpeg

This file provides the complete flight trajectory information presented in Figure 11. The file contains 7 columns as defined previously (data in Figure 8) and the background image is also provided

Figure12_Spatial distribution of mosquito activity.xls

This file includes the data presented in Figure 12: Spatial distribution of mosquito activity for *Cx. Quinquefasciatus.*
