# Supplementary figures and images for: A novel video-tracking system to quantify the behaviour of nocturnal mosquitoes attacking human hosts in the field
Source: J R Soc Interface. 2016 Apr;13(117):20150974. doi: 10.1098/rsif.2015.0974 (PMC4874425; doi:10.1098/rsif.2015.0974)

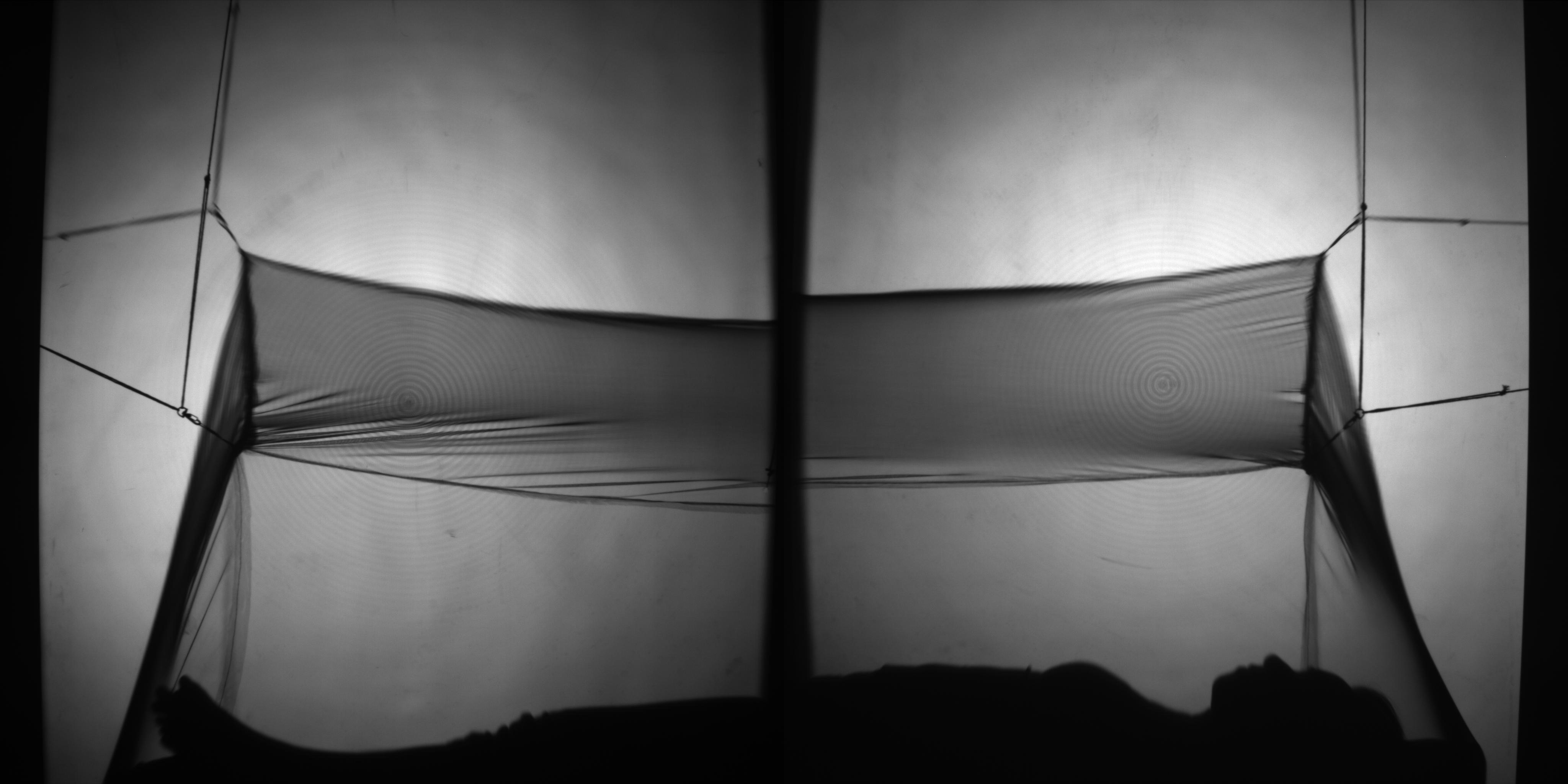

Supplement: Figure8_Image [file rsif20150974supp4.jpeg]

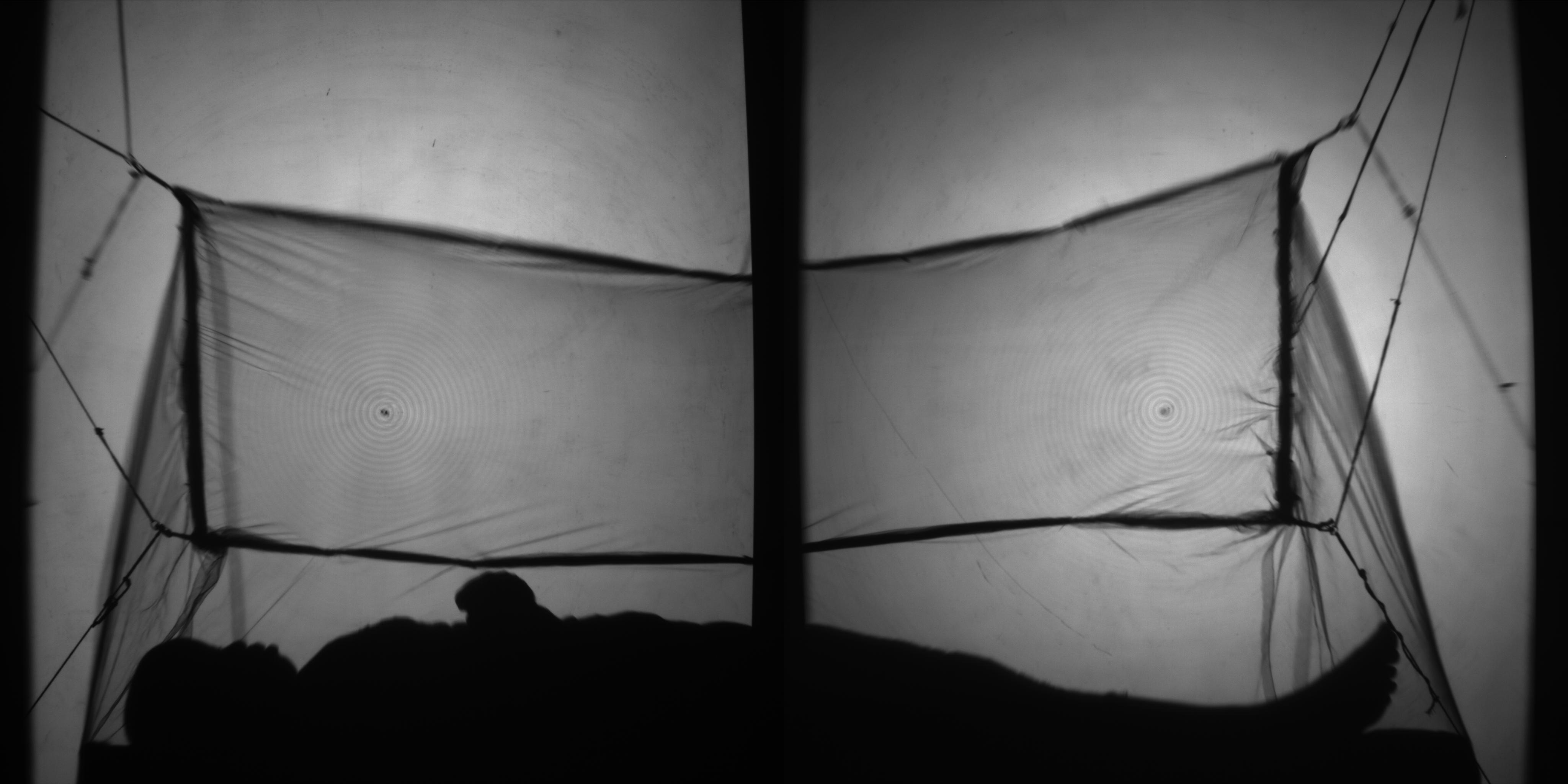

Supplement: Figure11_Image [file rsif20150974supp6.jpeg]
